# Single-cell RNA-Seq reveals the potential risk of anti-mesothelin CAR T Cell therapy toxicity to different organs in humans

**DOI:** 10.3389/fimmu.2022.807915

**Published:** 2022-08-17

**Authors:** Lu Wen, Yu Huang, Ling Peng, Kaiping Zhao, Yan Sun, Zhicai Lin, Yuanyuan Chen, Zhong Li, Qijun Qian, Fan Tong, Ruiguang Zhang, Xiaorong Dong

**Affiliations:** ^1^ Cancer Center, Union Hospital, Tongji Medical College, Huazhong University of Science and Technology, Wuhan, China; ^2^ Department of Medical Record Management and Statistics, Beijing Jishuitan Hospital, Beijing, China; ^3^ Shanghai Cell Therapy Group Corporation, Shanghai, China; ^4^ Mengchao Cancer Hospital, Shanghai University, Shanghai, China

**Keywords:** chimeric antigen receptor engineered T cells, mesothelin, single-cell RNA-seq, human organs, toxicity

## Abstract

“On-target off-tumor” toxicity is a major challenge to the use of chimeric antigen receptor (CAR)-engineered T cells in the treatment of solid malignancies, because of the expression of target antigens in normal tissues. Mesothelin overexpression is associated with poor prognosis of multiple solid tumors, and would therefore appear to be a suitable antigen target. To understand the risk of toxicity to different organs on anti-mesothelin CAR T cell therapy, single-cell RNA sequencing (scRNA-seq) datasets derived from major human physiological systems were analyzed in this study, including the respiratory, cardiovascular, digestive, and urinary systems. According to scRNA-seq datasets, the organs were stratified into high or low risk based on the level of mesothelin expression. We report that the proportion of mesothelin-positive cells was 7.71%, 2.40% and 2.20% of myocardial cells, pulmonary cells and stomach cells, respectively, indicating that these organs could be at high risk of “on-target off-tumor” toxicity on anti-mesothelin CAR T cell therapy. By contrast, esophagus, ileum, liver, kidney and bladder exhibited low mesothelin expression (<1%). Therefore, these organs could be regarded as at low risk. Thus, the risk of toxicity to different organs and tissues in anti-mesothelin CAR T cell therapy may be predicted by these scRNA-seq data.

## Introduction 

Immunotherapy is an important treatment for solid tumors and hematological malignancies. Chimeric antigen receptor (CAR) T cells are genetically engineered to recognize surface antigens independently of major histocompatibility complex restriction (1). CARs are introduced into T cells using viral or non-viral vectors for integrating genes, imbuing the CAR T cells with antigen-specific recognition ability, activation, proliferation, and cytotoxic function (2, 3). CAR T cell therapy targeting CD19 has achieved durable clinical responses in B-cell malignancies. However, CAR T cell therapy has been less effective against solid tumors. It is critical to identify ideal antigens to tackle solid tumors.

Mesothelin (MSLN) is a 40 kDa membrane protein anchored to the cell membrane by glycosylphosphatidylinositol (GPI) (4). MSLN is widely overexpressed in multiple solid cancers, such as ovarian, colorectal, pancreatic, and breast cancers (5–7). Aberrant MSLN expression plays an important role in tumor cell proliferation and invasion by inducing the activation of matrix metalloproteinase (MMP) (8, 9). AKT/PI3K/NF-κB pathways are activated by the overexpression of MSLN and subsequently induce resistance to apoptosis, which might help cancer cell survival in the highly inflammatory milieu (10). MSLN was selected as a target tumor antigen based on its association with poor prognosis and overexpression in various solid cancers (4). MSLN-CAR-T cell therapy decreased the growth of various MSLN-positive tumors and increased cytokine levels both *in vitro* and *in vivo* (5, 11).

However, “on-target off-tumor” activity is a major challenge for CAR T therapy because the target antigens are also expressed in normal tissues (12). Zhang et al found that MSLN was expressed by 55% and 63% of colorectal and ovarian cancers, respectively. By contrast, MSLN was expressed in 16% of the adjacent tissues in colorectal samples and 15% of non-cancerous normal ovaries (5). Therefore, it is critical to construct CAR T cells that target tumor tissues with negligible off-tumor toxicity. This study evaluated the potential risk of “on-target off-tumor” toxicity for different human organs in anti-MSLN CAR T cell therapy by single-cell RNA-seq data analysis.

## Materials and methods

The scRNA-seq data used in this study were mainly acquired from the Gene Expression Omnibus (GEO) database. The heart data were from GSE106118 (98 samples). The lung data were from GSE122960 sample GSM3489185 (1 sample). The stomach data were from GSE134520 sample GSM3954949 (1 sample). The ileum data were from GSE134809 sample GSM3972018 (1 sample). The liver data were from GSE115469 (5 samples). The kidney and the bladder data were from GSE131685 sample GSM4145205 (1 sample) and GSE129845 sample GSM3723358 (1 sample), respectively. The esophagus data were downloaded from https://www.tissuestabilitycellatlas.org/ (6 samples).

This study evaluated the MSLN expression distribution in distinct cell types of different organs, then the cell types with high MSLN expression levels were identified in accordance with the scRNA-seq datasets. Any type of cells with proportion (UMI count>0) of MSLN were defined as MSLN positive cells. We defined the cell types with a >1% proportion of MSLN positive cells as high risk and those with a <1% proportion MSLN positive cells as low risk.

We used 10x Genomics Chromium Single Cell 3’ Reagents Kit v2 user guide to perform single cell suspension. The proportion of MSLN expression was calculated using preliminary quality control data, the standard of quality control was that the gene expressed in at least 1 cell was reserved, and the cell expressing at least 100 genes was reserved. Seurat V3.0 was used to discriminate different cell types. The data were first normalized using the LogNormalize method, which was the expression value of each gene was divided by the expression value of all genes in the entire cell, multiplied by 10000, and then logarithmically transformed, then scale-shifted all genes to ensure that the mean and variance of each gene's expression in all cells were 0 and 1, and that each gene had the same weight in the downstream analysis, rather than the HVG playing a dominant role, the cell clustering performance were conducted using the top 2000 most variable genes. We used the UMAP method to obtain cell scatter plots (13).

We performed dimensionality reduction with principal component analysis (PCA) on the sample for each tissue. Once embedded in this PCA space, a nearest neighbor graph identifying the k=10 nearest neighbors for each cell was constructed. We derived UMAP embeddings presented for visualization from this nearest neighbor graph using a minimum distance of 0.5. SingleR (Single-cell Recognition of cell types) was used to identify the cell type of each cluster (http://www.bioconductor.org/packages/release/bioc/vignettes/SingleR/inst/doc/SingleR.html).

To test MSLN protein expression, different tissues were cut to the optima size and immersed in frozen solution, or embedded in paraffin and sectioned in 6μm, and mounted on slides. After endogenous biotin blocker and goat serum incubation, slides were incubated with alpaca anti-MSLN-VHH-biotin (1:100) at 4°C overnight, followed by Streptavidin-HRP for 30 min. The sections were developed with freshly prepared solution containing 3,3’-diaminobenzidine and counterstained with hematoxylin. Images were obtained on a Zeiss Axiophot microscope and analyzed in the AxioVision software (Zeiss).

## Results

### Myocardial cells exhibit high mesothelin expression

**Figure 1:**
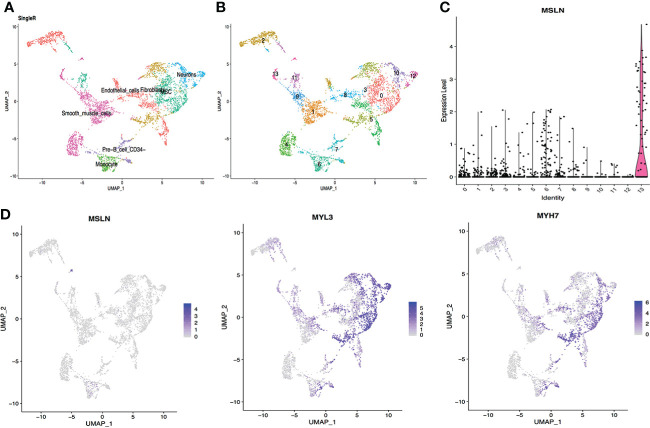
Analysis of cardiac scRNA-seq data showing high expression of MSLN in myocardial cells. **(A)** Cell types in the heart according to SingleR. **(B)** Cells in the heart were categorized into 14 different clusters, numbered 0 to 13. **(C)** Violin plot of MSLN expression distribution in these 14 different cell clusters. **(D)** Scatter plots showing that the cluster of cells with high expression of MSLN also expressed the typical myocardial cells markers MYL3 and MYH7.

Myocardial cells were categorized into 14 different clusters according to the scRNA-seq data, including smooth muscle cells, endothelial cells, fibroblasts, monocyte, neurons etc. (**Figures 1A**, **B**). The distribution of MSLN expression in these 14 different cell clusters is shown as violin plots in **Figure 1C** and scatter plots in **Figure 1D**. This showed that 7.71% of myocardial cells expressed MSLN, indicating that there may be a high risk of “on-target off-tumor” toxicity in the heart for MSLN-CAR-T cell therapy. MSLN was mainly expressed in smooth muscle cells (cluster 13) according to SingleR (**Figure 1** and **Supplementary Table S1**). Expression of the typical markers of myocardial cells MYL3 and MYH7 was confirmed in these cell clusters (**Figure 1D**).

### Pulmonary cells show high mesothelin expression

**Figure 2:**
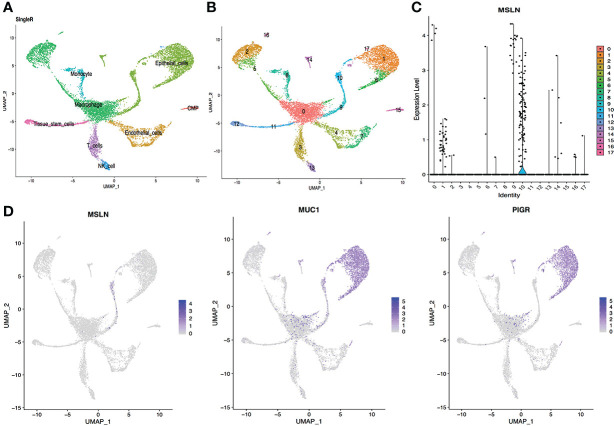
scRNA-seq data analysis of lung showing pulmonary cells with high MSLN expression. **(A)** Cell types in the lung according to SingleR. **(B)** Cells in the lung were categorized into 18 different clusters, numbered 0 to 17. **(C)** Violin plot of MSLN expression distribution in these 18 different cell clusters. **(D)** Scatter plots showing thagt the cluster of cells with high expression of MSLN also expressed the typical pulmonary cells markers MUC1 and PIGR.

The scRNA-seq data from respiratory system tissues indicated that pulmonary cells contained approximately 2.40% MSLN-positive cells, so the lung could be at high risk of “on-target off-tumor” toxicity for MSLN-CAR-T cell therapy. The pulmonary cells were classified into 18 different clusters and MSLN expression distribution in these 18 cell clusters is shown in **Figure 2**. The cluster of cells mainly expressing MSLN was composed of epithelial cells (**Figure 2** and **Supplementary Table S1**). The typical markers of pulmonary cells MUC1 and PIGR were confirmed as present in these cells (**Figure 2D**).

### Stomach has high MSLN expression

**Figure 3:**
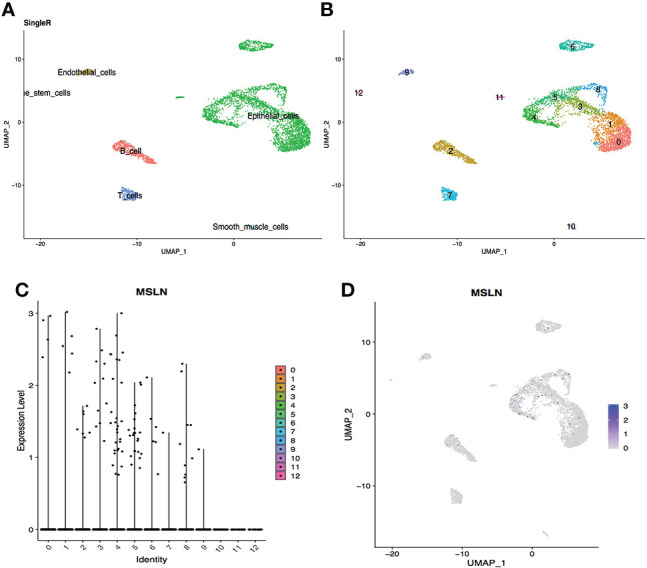
Analysis of scRNA-seq data from stomach cells showing high expression level of MSLN. **(A)** Cell types in the stomach according to SingleR. **(B)** Cells in the stomach were categorized into 13 different clusters, numbered 0 to 12. **(C)** Violin plot of MSLN expression distribution in these 13 different cell clusters. **(D)** MSLN expression distribution shown as a scatter plot.

**Figure 4:**
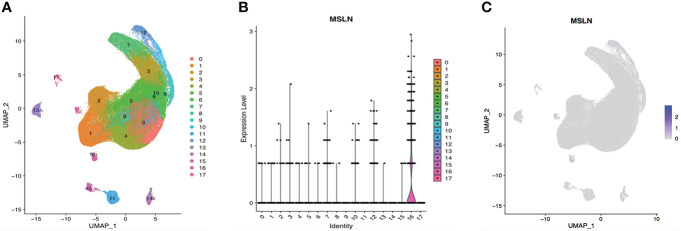
ScRNA-seq data analysis showing esophageal cells with low MSLN expression. **(A)** Cells in the esophagus were categorized into 18 different clusters, and numbered 0 to 17. **(B)** Violin plot of MSLN expression distribution in these 18 different cell clusters. **(C)** MSLN expression distribution shown as a scatter plot.

The scRNA-seq datasets from the digestive system were explored, including stomach, esophagus, ileum, and liver, showing 2.20% cells from the stomach were MSLN-positive (**Figure 3**). This suggest that the stomach could also be at high risk. MSLN was mainly expressed in epithelial cells of the stomach (**Figure 3** and **Supplementary Table S1**). By contrast, the proportion of MSLN-positive cells was only 0.67%, 0.34% and 0.25% in esophageal epithelial cells, ileal epithelial cells and hepatic cells, respectively (**Figures 4** **–** **6**). Therefore, esophagus, ileum, and liver could be regarded as low risk. The scatter plots for cell markers of stomach, esophagus, and liver were not available in the scRNA-seq datasets.

**Figure 5:**
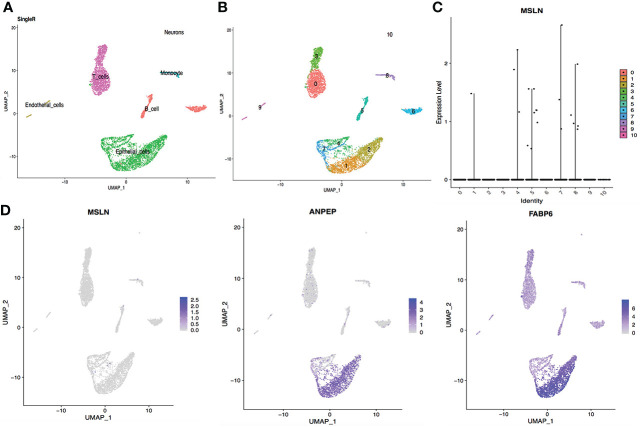
Analysis of scRNA-seq data from ileal cells showing low MSLN expression. **(A)** Cell types in the ileum according to SingleR. **(B)** Cells in the ileum were categorized into 11 different clusters, numbered 0 to 10. **(C)** Violin plot of MSLN expression distribution in these 11 different cell clusters. **(D)** Scatter plots showing the cluster of cells with MSLN expression also expressed the typical ileal cells markers ANPEP and FABP6.

**Figure 6:**
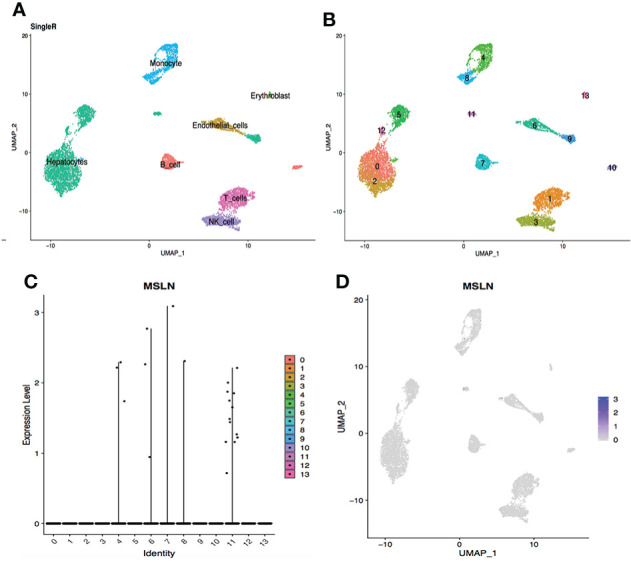
scRNA-seq data analysis of hepatic cells showing low MSLN expression. **(A)** Cell types in the liver according to SingleR. **(B)** Cells in the liver were categorized into 14 different clusters, numberedd 0 to 13. **(C)** Violin plot of MSLN expression distribution in these 14 different cell clusters. **(D)** The MSLN expression distribution shown as a scatter plot.

### Kidney and bladder cells

**Figure 7:**
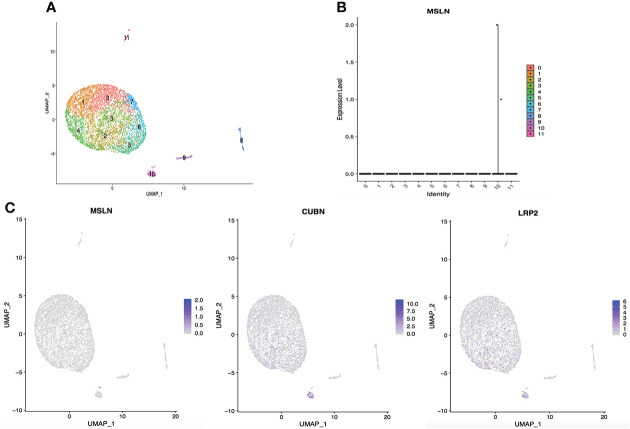
Analysis of scRNA-seq data showing low expression of MSLN in the kidney. **(A)** Cells in the kidney were categorized into 12 different clusters, numbered 0 to 11. **(B)** Violin plot of MSLN expression distribution in these 12 different cell clusters. **(C)** Scatter plots showing that the cluster of cells with MSLN expression also expressed the typical renal cells markers CUBN and LRP2.

**Figure 8:**
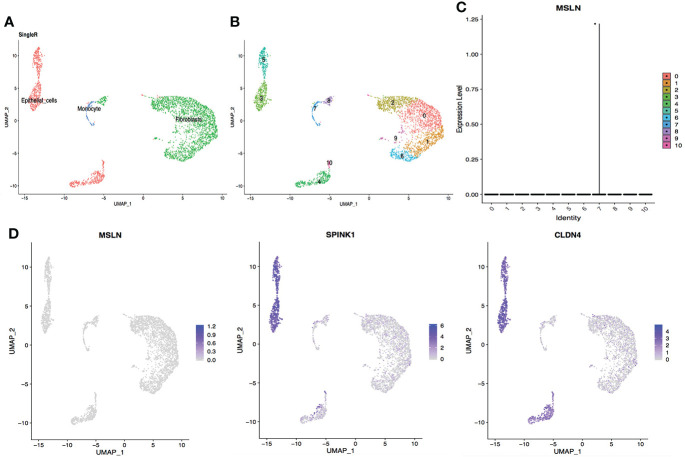
ScRNA-seq data analysis showing bladder urothelial cells with low MSLN expression. **(A)** Cell types in the bladder according to SingleR. **(B)** Cells in the bladder were categorized into 11 different clusters, numbered 0 to 10. **(C)** Violin plot of MSLN expression distribution in these 11 different cell clusters. **(D)** Scatter plots showing that the cluster of cells with MSLN expression also expressed the typical bladder urothelial cells markers SPINK1 and CLDN4.

The scRNA-seq data of cells in the urinary system were also analyzed, including kidney and bladder. The cells in kidney were categorized into 12 clusters and 0.05% cells showed MSLN expression (**Figure 7**). In addition, only 0.03% MSLN-positive cells were found in the bladder (**Figure 8**). Kidney and bladder are therefore at low risk of “on-target off-tumor” toxicity for MSLN-CAR-T cell therapy.

**Figure 9:**
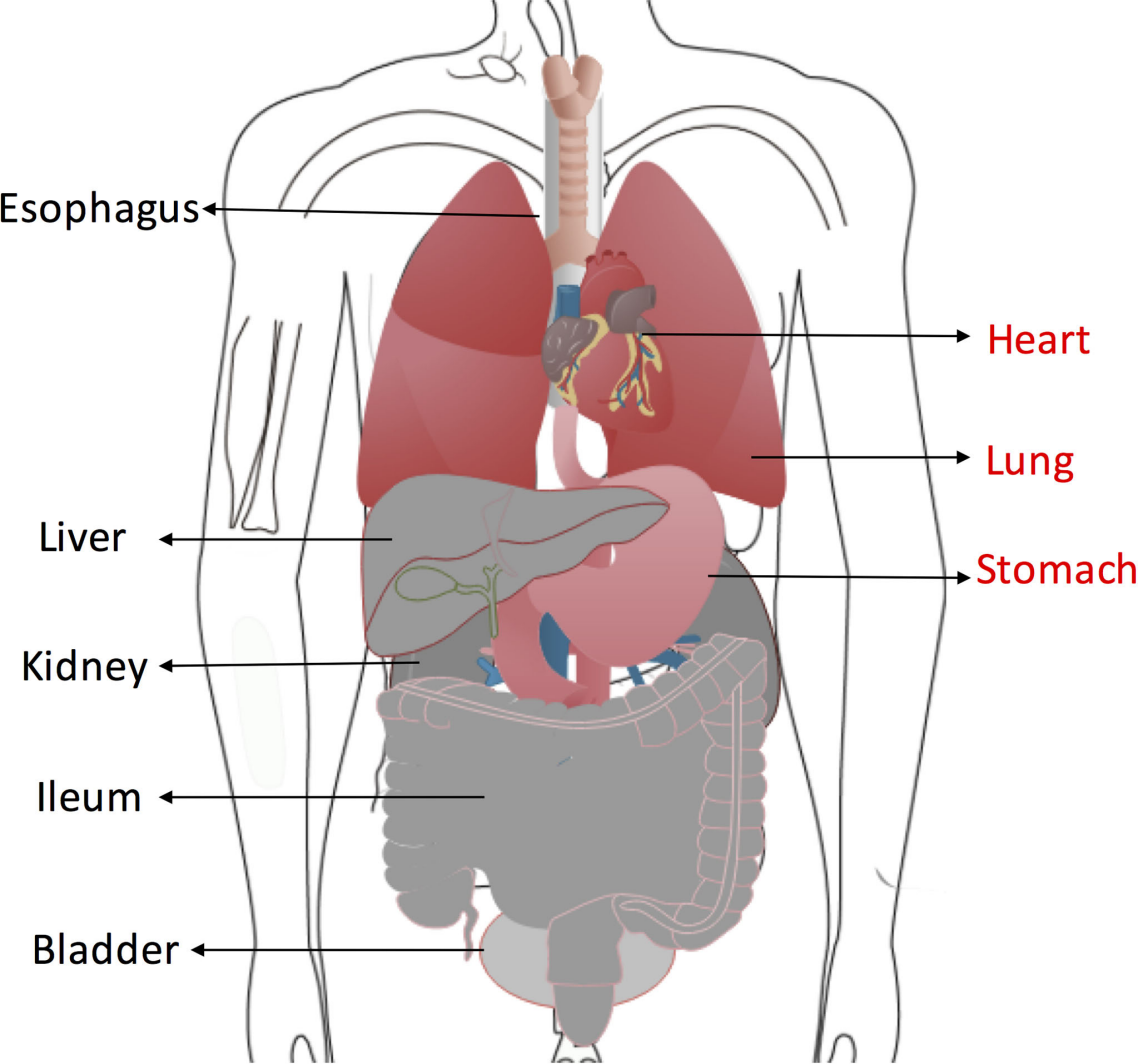
Organs at high risk of “on-target off-tumor” toxicity for anti-MSLN CAR T cells therapy are highlighted in red. Low-risk organs including esophagus, ileum, liver, kidney and bladder are shown in gray.

According to the above results, a potential risk map of “on-target off-tumor” toxicity for different organs was constructed (**Figure 9**). Myocardial cells were found to have the highest MSLN expression, followed by pulmonary cells and stomach cells. Other organs showed low MSLN expression.

### MSLN protein expression

Seven tissues have been tested for affinity of anti-MSLN-VHH assay (**Supplementary Figure S1**). Anti-MSLN was used as the positive control for MSLN expression. Anti-MSLN-VHH was generated in our lab and used to detect proteins binding to this nanoantibody. The results show that anti-MSLN can have a wide range of positive reactions in fibrous structure areas and a weak positive reaction with epithelial cells of a small number of organs. Anti-MSLN-VHH weakly binds to fibrous structures. Both antibodies were negative in liver, kidney, and heart (**Supplementary Table S2**).

## Discussion

CD19-targeted CAR T cell therapies can achieve durable clinical responses in B-cell malignancies (14). However, CAR T cell therapy is also associated with toxicities such as cytokine release syndrome (CRS), neurotoxicity or “on-target off-tumor” effects (14, 15). CD133-CAR-T cells have a major drawback for CD133 as target in immunotherapy because its expression in hematopoietic stem and progenitor cells (HSPCs), which would likely exert “on-target off-tumor” myeloablative toxicity (16). Carboxyanhydrase-IX-specific CAR T cells might cause the cholestasis due to expression of carboxyanhydrase-IX on bile duct epithelium (17). Fatal respiratory failure and multiorgan dysfunction was also reported in a patient with colon cancer treated with HER2-specific CAR T cells, resulting from expression of the target antigen in lung tissue (18). This possibility of “on-target off-tumor” toxicity is a great obstacle for the successful use of CAR T cell therapies in solid tumors.

MSLN is a potential target for cancer immunotherapy, considering its low expression on normal cells and high expression in a variety of solid malignancies. Previous studies indicate that aberrant MSLN expression promotes cancer cell proliferation, local invasion and metastasis (8, 19). Anti-MSLN CAR T cells have been evaluated in several preclinical models and clinical trials. Beatty et al showed that adoptive transfer of mRNA CAR T cells targeting MSLN is feasible and safe in two case reports (20). No “on-target off-tumor” toxicity against normal tissues was observed (20). In a phase I study, 6 patients with chemotherapy-refractory metastatic pancreatic ductal adenocarcinoma received autologous MSLN-specific CAR T cells; none developed CRS or neurologic events and there were no dose-limiting toxicities (21). Another phase I study investigated the safety and activity of lentiviral-transduced autologous MSLN-specific CAR T cells in 15 patients with malignant pleural mesothelioma, ovarian carcinoma and pancreatic ductal adenocarcinoma. The best overall response was stable disease and one dose-limiting toxicity of grade 4 sepsis occurred (22). In addition, anaphylaxis and cardiac arrest was reported within minutes of completing the third infusion of anti-MSLN CAR T cells in one patient (23).

Due to the small sample size of previous studies, anti-MSLN CAR T treatment for solid tumors is still in the exploratory stage, and its potential adverse effects are not fully understood. Normal expression of MSLN is thought to be restricted primarily to the mesothelial cells of the pleura, pericardium, peritoneum, and tunica vaginalis in men according to previous studies (12). In the present study, scRNA-seq datasets of different human organs were analyzed to identify potential “on-target off-tumor” toxicity. Data analysis for each organ was independent. The cells in each organ were divided into different clusters and MSLN expression of these cell clusters was explored. High MSLN expression was found in heart, lung and stomach, indicating that these organs could be at high risk of “on-target off-tumor” toxicity for anti-MSLN CAR T cell therapy. By contrast, esophagus, ileum, liver, kidney and bladder showed low MSLN expression and could be regarded as low risk. MSLN expression might vary between individuals and only one sample in each organ of lung, stomach, ileum, kidney and bladder was available for scRNA-seq data analysis. Low MSLN expression of ileum, kidney and bladder should be explored with more samples. In addition to including more samples, sampling cells from organs within the same individual will control for confounding variables. SingleR was used to identify cell types of different clusters for these organs, showing that MSLN was mainly expressed in smooth muscle cells of the heart and epithelial cells in lung and stomach. The cell types for different clusters in the esophagus were not available according to SingleR.

To identify MSLN protein expression in different organs, we applied immunohistochemistry and found that anti-MSLN-VHH staining of lung, stomach, liver and kidney was consistent with the scRNA-seq datasets. The level of MSLN protein in each organ was explored in single sample of human tissue. Because of the variation between individuals, in certain cases, inconsistent MSLN protein expression of heart, ileum and bladder with scRNA-seq datasets still needs validation in more samples. Esophageal tissue samples were not available, so the protein expression for this organ could not be established. Further basic research and clinical trials are still needed to explore the toxicity of anti-MSLN CAR T cell therapy for different organs.

CAR T cells may cause “on-target off-tumor” toxicity through their recognition of healthy cells that express the target antigen. Previous data focusing on the toxicity of anti-MSLN CAR T cell therapy are limited. The present study evaluated the potential risk of toxicity for different human organs of anti-MSLN CAR T cell therapy by single-cell RNA-seq data analysis. The organs could be stratified into high or low risk according to MSLN expression, which might provide potential clues for further investigation.

## Data availability statement

The datasets presented in this study can be found in online repositories. The names of the repository/repositories and accession number(s) can be found in the article/**Supplementary Material**.

## Author contributions

LW and XD conceived and designed the research. LW, YH and LP interpreted the data and wrote the manuscript. KZ collected the data and performed all analysis. YS, ZCL, YC, ZL and QQ interpreted the data, reviewed and edited the manuscript. All authors contributed to the article and approved the final version of the manuscript.

## Funding

This research was supported by the grants from the National Key R&D Program of China (Grant No 2019YFC1316205).

## Conflict of interest

Authors YS, ZCL, YC, ZL, and QQ are employed by Shanghai Cell Therapy Group Corporation.

The remaining authors declare that the research was conducted in the absence of any commercial or financial relationships that could be construed as a potential conflict of interest.

## Publisher’s note

All claims expressed in this article are solely those of the authors and do not necessarily represent those of their affiliated organizations, or those of the publisher, the editors and the reviewers. Any product that may be evaluated in this article, or claim that may be made by its manufacturer, is not guaranteed or endorsed by the publisher.

## Supplementary material

Supplementary Figure 1 Affinity of anti-MSLN-VHH to human tissues. A. Positive staining of MSLN in the selected tissues (staining brown): stomach, ileum, lung and bladder. B. Negative staining of MSLN in the selected tissues: heart, liver, kidney.
