# Supplementary figures and images for: Single-cell RNA-Seq reveals the potential risk of anti-mesothelin CAR T Cell therapy toxicity to different organs in humans
Source: Front Immunol. 2022 Aug 17;13:807915. doi: 10.3389/fimmu.2022.807915 (PMC9428152; doi:10.3389/fimmu.2022.807915)

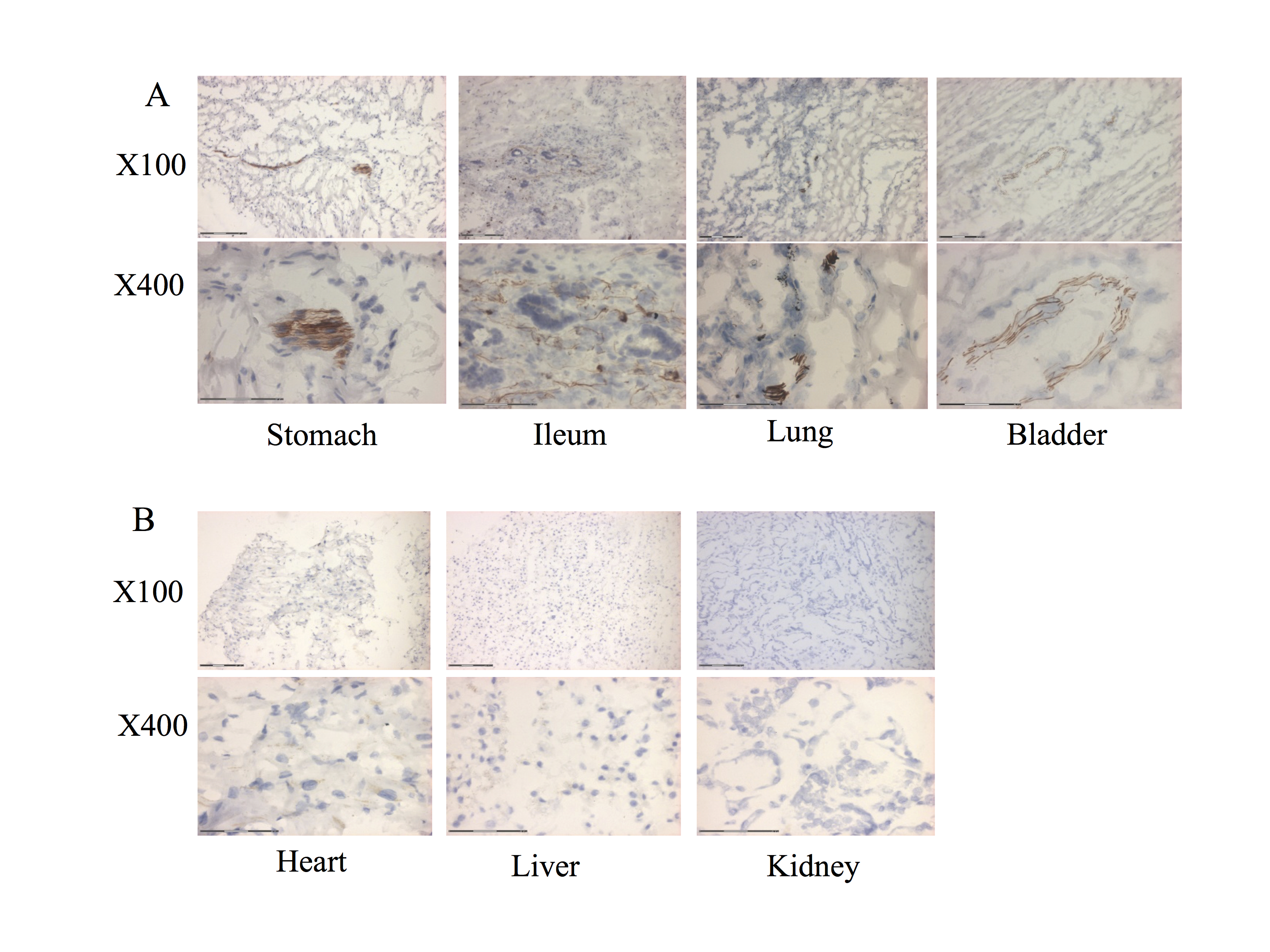

Supplement: Supplementary Figure 1 — Affinity of anti-MSLN-VHH to human tissues. A. Positive staining of MSLN in the selected tissues (staining brown): stomach, ileum, lung and bladder. B. Negative staining of MSLN in the selected tissues: heart, liver, kidney. [file Image_1.jpeg]
