# Supplementary material for: Single-cell RNA-Seq reveals the potential risk of anti-mesothelin CAR T Cell therapy toxicity to different organs in humans
Source: Front Immunol. 2022 Aug 17;13:807915. doi: 10.3389/fimmu.2022.807915 (PMC9428152; doi:10.3389/fimmu.2022.807915)
Supplement: Supplementary file 2 [file Table_1.docx]

Supplementary Table 1. Cell types of different clusters in each organ.

| Cluster ID | Heart | Lung | Stomach | Ileum | Liver | Bladder |
| --- | --- | --- | --- | --- | --- | --- |
| 0 | MSC | Macrophage | Epithelial cells | T cells | Hepatocytes | Fibroblasts |
| 1 | Smooth muscle cells | Epithelial cells | Epithelial cells | Epithelial cells | T cells | Fibroblasts |
| 2 | Endothelial cells | Macrophage | B cell | Epithelial cells | Hepatocytes | Fibroblasts |
| 3 | Fibroblasts | T cells | Epithelial cells | T cells | NK cell | Epithelial cells |
| 4 | Smooth muscle cells | Endothelial cells | Epithelial cells | Epithelial cells | Monocyte | Epithelial cells |
| 5 | Endothelial cells | Macrophage | Epithelial cells | B cell | Hepatocytes | Epithelial cells |
| 6 | Monocyte | Epithelial cells | Epithelial cells | B cell | Endothelial cells | Fibroblasts |
| 7 | Pre-B cell CD34- | Endothelial cells | T cells | Epithelial cells | B cell | Monocyte |
| 8 | Endothelial cells | Monocyte | Epithelial cells | Monocyte | Monocyte | Fibroblasts |
| 9 | Smooth muscle cells | Epithelial cells | Endothelial cells | Endothelial cells | Hepatocytes | Fibroblasts |
| 10 | Neurons | Epithelial cells | Smooth muscle cells | Neurons | B cell | Epithelial cells |
| 11 | MSC | Tissue stem cells | Epithelial cells |  | Hepatocytes |  |
| 12 | Neurons | Tissue stem cells | Tissue stem cells |  | Hepatocytes |  |
| 13 | Smooth muscle cells | NK cell |  |  | Erythroblast |  |
| 14 |  | Epithelial cells |  |  |  |  |
| 15 |  | CMP |  |  |  |  |
| 16 |  | Macrophage |  |  |  |  |
| 17 |  | NK cell |  |  |  |  |
