# Supplementary material for: Single-cell RNA-Seq reveals the potential risk of anti-mesothelin CAR T Cell therapy toxicity to different organs in humans
Source: Front Immunol. 2022 Aug 17;13:807915. doi: 10.3389/fimmu.2022.807915 (PMC9428152; doi:10.3389/fimmu.2022.807915)
Supplement: Supplementary file 3 [file Table_2.docx]

Supplementary Table 2. The potential MSLN protein expression

| Tissues | Positive control | Negative | anti-MSLN |
| --- | --- | --- | --- |
| Lung | Alveolar epithelium is positive (2%) | - | Fibrous structure |
| Stomach | Gastric mucosal epithelium is positive (5%) | - | Fibrous structures in the submucosal matrix |
| Ileum | Positive in envelope | - | Fibrous structure in the muscle layer |
| Bladder | Migrating epithelium positive (2%) | - | Fibrous structure in matrix |
| Liver | - | - | - |
| Kidney | - | - | - |
| Heart | - | - | - |
